# DCLK1 Inhibition Sensitizes Colorectal Cancer Cells to Radiation Treatment

**DOI:** 10.22088/IJMCM.BUMS.10.1.23

**Published:** 2021-05-22

**Authors:** Chiman Mohammadi, Ali Mahdavinezhad, Massoud Saidijam, Fatemeh Bahreini, Abdolazim Sedighi Pashaki, Mohammad Hadi Gholami, Rezvan Najafi

**Affiliations:** 1 *Research Center for Molecular Medicine, Hamadan University of Medical Sciences, Hamadan, Iran.*; 2 *Mahdieh Radiotherapy and Brachytherapy Charitable Center, Hamadan, Iran.*

**Keywords:** DCLK1, ionizing radiation, colorectal cancer, radiosensitivity

## Abstract

Colorectal cancer (CRC) is one of the most prevalent diagnosed cancers and a common cause of cancer-related mortality. Despite effective clinical responses, a large proportion of patients undergo resistance to radiation therapy. Therefore, the identification of efficient targeted therapy strategies would be beneficial to overcome cancer radioresistance. Doublecortin-like kinase 1 (DCLK1) is an intestinal and pancreatic stem cell marker that showed overexpression in a variety of cancers. The transfection of *DCLK1* siRNA to ‎normal HCT-116 cells was performed, and then cells were irradiated with X-rays. The effects of *DCLK1* inhibition on cell survival, apoptosis, cell cycle, DNA damage response (ATM and γH2AX proteins), epithelial-mesenchymal transition (EMT) related genes (vimentin, N‐cadherin, and E-cadherin), cancer stem cells markers (*CD44*, *CD133*, *ALDH1*, and *BMI1*), and β‐catenin signaling pathway (β‐catenin) were evaluated. *DCLK1 *siRNA downregulated *DCLK1* expression in HCT-116 cells at both mRNA and protein levels (P* <*0.01). Colony formation assay showed a significantly reduced cell survival in the *DCLK1* siRNA transfected group in comparison with the control group following exposure to 4 and 6 Gy doses of irradiation (P <0.01). Moreover, the expression of cancer stem cells markers (P <0.01), EMT related genes (P <0.01), and DNA repair proteins including pATM (P <0.01) and γH2AX (P <0.001) were significantly decreased in the transfected cells in comparison with the nontransfected group after radiation. Finally, the cell apoptosis rate (P <0.01) and the number of cells in the G0/G1 phase in the silencing *DCLK1* group was increased (P <0.01). These findings suggest that *DCLK1 *can be considered a promising therapeutic target for the treatment of radioresistant human CRC.

Dolorectal cancer (CRC) is considered the third leading cause of cancer-related death for both men and women worldwide (1). Radiotherapy (RT) as a highly effective treatment approach for cancer has been broadly used in the clinic for over 100 years, where directly or indirectly mediating tumor cell death via inducing multiple types of DNA damage and genome instability (2). Indeed, RT improves the efficacy of surgery by shrinking the tumor before surgery or removing the remaining microscopic tumor cells afterward (3). Despite promising achievement in radiation therapy for controling malignant cells, and curative potentials in several localized cancers, prognosis and the 5-year survival rate remained poor, largely due to intrinsic cellular resistance (4). Therefore, in order to enhance the efficacy of RT, more therapeutic strategies are required for promoting the ionizing radiation (IR) sensitivity of CRC and to overcome IR resistance (5).

A growing body of evidence reveals that various factors including double-strand break (DSB) repair pathway through homologous recombination and nonhomologous end-joining, tumor microenvironment, various deregulated signaling pathways (e.g., AKT or NF-‎κB,), micro RNA dysregulation, redistribution of ‎the cell cycle, hypoxia, and apoptosis contribute ‎to cellular resistance against radiation (6, 7). Besides, cancer stem cells (CSCs) or tumor-initiating cells, and the epithelial-mesenchymal transition (EMT) enable radioresistant tumor cells metastasis after IR exposure (8). Discovering and targeting molecules and signaling pathways associated with these barriers are essential for developing new beneficial radiosensitizers, and improving the efficacy ofIR (9).

Doublecortin-like kinase 1 (DCLK1) as a tuft cell marker in the small intestine is a member of the microtubule-associated protein kinase superfamily that regulates embryonic cortical development and neuronal process (10). Cumulative evidence has verified that *DCLK1* expression can support CSCs self-renewal, cancer growth, EMT, and metastasis in both early and advanced cancer stages (11). Moreover, EMT and CSCs that play key roles in the development and progression of cancer, are involved in multimodal therapy resistance and relapse (12).

Here, we hypothesized that IR-induced ataxia telangiectasia mutated (ATM) activation following direct interaction with DCLK1 may lead to the repair of damaged DNA, and increase the survival of cancer cells. Therefore, the present study aimed to explore whether *DCLK1* inhibition impacts radiosensitivity, double strand breack (DSB) repair, cell cycle, cell survival, EMT, and CSCs expression in HCT-116 CRC cell line, and elucidatethe underlying mechanisms through *in vitro* investigations.

## Materials and methods

**Cell culture and transfection**

The human HCT-116 CRC cell line was obtained from the National Cell Bank, Pasteur Institute, Tehran, Iran (http://fa.pasteur.ac.ir/), and radioresistant cells HCT-116 (RR−HCT-116) were generated by fractional radiation (13). The cells were maintained in Dulbecco’s modified Eagle’s medium (DMEM; Gibco, USA) supplemented with 10% fetal bovine serum (FBS; Gibco, USA), and 1% penicillin or streptomycin, thereafter incubated in a humidified atmosphere of 5% CO_2_ at 37 °C. The sequence of siRNA against *DCLK1* (accession No. NM_001195415) (5'GGUUUAUAACUUCGA CACATT3') and HiPerFect siRNA transfection reagent were obtained from QIAGEN (Hilden, Germany). To perform transfection, HCT-116 cells were seeded into six-well plates at a density of 3×10^5^ cells per well with no antibiotic and FBS added. When the cells reached ~60–70% confluency, they were transfected with siRNA (5 nM) using 12 μL of the transfection reagent, following the manufacturer’s protocol (QIAGEN, Hilden, Germany). 

**Colony** **‐** **forming assay**

IR was delivered using a synergy linear accelerator with an agility collimating device (Elekta AB, Stockholm, Sweden, 200 MU/min dose rate) at the specified dose. The cells were counted and replated in 6-well plates at 24 h after transfection, then incubated for an additional 24 h. Next, the cells were exposed to IR in a single fraction, incubated for seven days, followed by staining with crystal violet. Using Olympus CKX53 inverted microscope (Nagano Olympus Co. Japan), the colonies (>50 cells) were quantified, and to obtain the average colony formation rate the experiment was repeated in triplicate. The following formula was used: plating efficiency (PE)= colony number/seeded cells number ×100%; survival fraction (SF)= the number of colonies formed by irradiated cells in response to a particular dose/seeded cells number × PE.

**Flow cytometry** **‐** **based cell** **‐** **cycle and apoptosis analysis**

Cell cycle analysis was carried out by quantification of DNA content staining through propidium iodide (PI; Abcam, USA), according to the manufacturer’s instructions. HCT-116 cells in the exponential growth phase were plated in six-well plates and transfected as mentioned earlier. The cells were collected and washed twice with phosphate-buffered saline (PBS) at 48 h post-exposure to 6 Gy radiation, then fixed with 70% ethanol at 4°C overnight. The cells incubation was conducted with DNAse-free RNAse (Sigma-Aldrich, Taufkirchen, Germany) at room temperature in the dark condition for 30 min and later stained with PI. The cell cycle status was evaluated by flow cytometry.

To analysis the rate of apoptosis, HCT-116 cells were grown in six-well plates, transfected and treated as mentioned earlier. Forty-eight hours after radiation, the cells stained with annexin V and PI were assessed by Attune NxT acoustic focusing cytometer (Invitrogen, USA). All the results were analyzed using Flowjo 7.6 software. (FlowJo, LLC, Ashland, OR, USA). Each experiment was repeated three times.

**RNA isolation and reverse-transcription quantit-ative PCR (RT** **‐** **qPCR)**

Per the manufacturer’s instructions, total RNA was isolated from HCT-116 cells using RNX‐Plus Kit (Cinnagen, Tehran, Iran). RNA quantity and quality were evaluated using a NanoDrop Spectrophotometer (Thermo Fisher Scientific, USA). The complementary DNA (cDNA) was synthesized using PrimeScript™ RT reagent Kit (TaKaRa, Japan) according to the manufacturer’s protocol. A master mix (10 μL in total) containing 5X PrimeScript Buffer (2 μL), RT mix (0.5 μL), oligo dT primer (0.5 μL), random hexamer primer (0.5 μL), 500 ng of total RNA (2-4 μL), and nuclease-free water (2.5-4.5 μL) was prepared on ice. The reaction was incubated at 37^o^C for 15 min, 85^o^C for 5 s, and at 4^o^C for 5 min. The RT-qPCR was conducted to assess the mRNA expression of 

**Table 1:** Primer sequences for qRT-PCR

**Reverse**	**Forward**	**Gene**
CAGGAAGGTCTCATTGAACAC	TTGCTCCAGATCGTTAGAAGG	*DCLK1*
AACGCCTTGTCCTTGGTAG	GAGTCGGAAACTGGCAGATAG	*CD133*
GAAGTTGCTGATGACCCATTTAC	CATCCACAGTTTCCTCACATTTC	*BMI1*
GCCCTTCTATGAACCCATACC	AATGGTCGCTACAGCATCTC	*CD44*
CTCGGAAGCATCCATAGTACG	GCCAGGTAGAAGAAGGAGATAAG	*ALDH1*
GAGGATGGTGTAAGCGATGG	AGAACGCATTGCCACATACA	*E-cadherin*
CGTTGATAACCTGTCCATC	CATTGAGATTGCCACCTAC	*Vimentin*
CCCACAATCCTGTCCACATC	ATTCGGGTAATCCTCCCAAATC	*N-cadherin*
CCTTCCATCCCTTCCTGTTTAG	CTTCACCTGACAGATCCAAGTC	*β* *‐* *catenin*
TTGACGGTGCCATGGAATTT	GCCATCAATGACCCC-TTCATT	*GAPDH*

the target genes (Table 1) in samples. For each PCR reaction, 1 μL of cDNA was added to SYBR Premix Ex Taq II Master Mix (Takara, Japan), PCR primers (10 pmol), and nuclease-free water in a total volume of 10 μL. The cycling program was performed as follows: initial denaturation at 95°C followed by 40 cycles of denaturation for 5 s at 95°C, annealing for 30 s at 60°C, extension for 30 s at 72°C. RT-qPCR assays were performed in triplicate, and the mean values were used to calculate mRNA expression. Glyceraldehyde 3‐phosphate dehydrogenase (*GAPDH*) was applied as an internal control gene. The differential expression of genes was calculated by the 2^−ΔΔCT^ method.

**Western blot analysis**

Cells were harvested at 72 h after transfection (i.e. 48 h post-exposure to 6 Gy radiation), and total protein was isolated using RIPA lysis buffer (Santa Cruz Biotechnology, Dallas, USA) containing protease and phosphatase inhibitors. The protein concentration was quantified by Bradford protein assay (14). Equal amounts of proteins (~50 μg) were separated on 10% SDS-PAGE and transferred onto nitrocellulose. Each sample was loaded three times. Non-specific binding was blocked by membrane incubation in 5% skimmed milk (Merck, Darmstadt, Germany) and membranes were probed overnight at 4°C with the following primary antibodies: Anti-DCLK1 (1:5000, Abcam, USA), anti-GAPDH (1:10000, Abcam, USA), anti phospho ATM (ser^1981^) (pATM) (1:50000, Abcam, USA), anti γH2AX (1:1000, Abcam, USA). The secondary horseradish peroxidase-conjugated goat antirabbit antibody was used to incubate the membrane for 1 h at room temperature, and immunoreactivity was detected using an enhanced chemiluminescence detection kit (Amersham, USA). The band intensity was digitized and quantified by ImageJ software (National Institute of Health, Bethesda, MD, USA).

**Statistical analysis**

Analysis of the data between different treatment groups was conducted using the SPSS16.0 software, and the data were compared by analysis of variance (one-way ANOVA). Alpha was set at 0.05. Values are expressed as mean ± standard error.

## Results

***DCLK1*** **expression is upregulated in RR−HCT-116** **‎**

**Fig. 1:**
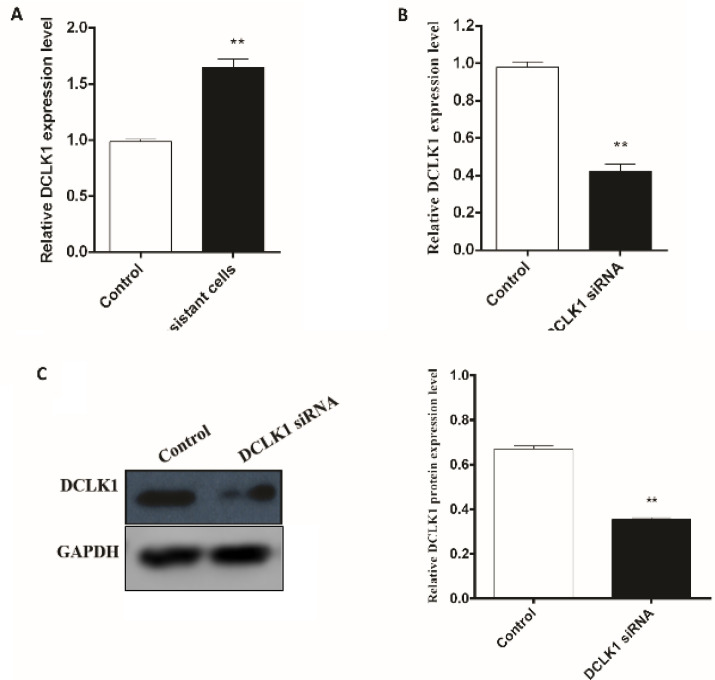
DCLK1 expression in radioresistant HCT-116 cells (RR-HCT-116) and effect of DCLK1 siRNA treatment on DCLK1 mRNA and protein expression. The expression levels of DCLK1 in RR-HCT-116 ‎(A) and transfected cells (B) determined by RT-qPCR 48 h after 6 Gy radiation. (C) Western blot analysis was performed to assess protein expression of DCLK1 in transfected HCT-116 with DCLK1 siRNA and nontransfected cells 48 h after 6 Gy radiation. All quantitative data are expressed as means ± SEM of three independent experiments. **P < 0. 01 vs. control

To investigate the effects of DCLK1 inhibition on radioresistance in colorectal cancer cells, the expression of this gene in radioresistant and normal cells was evaluated. The results showed an upregulation of *DCLK1* in radioresistant cells in comparison with normal cells (P <0.01; Figure 1A).

***DCLK1*** **siRNA downregulates** ***DCLK1*** **mRNA and protein expression**

Following *DCLK1* expression association with radioresistance evaluation, the cells were transfected with *DCLK1* siRNA, and were irradiated after 24 h. To detect the effect of siRNA-*DCLK1*, we examined *DCLK1* gene and protein expression in the CRC cells by RT-qPCR and western blotting analysis. In the transfected HCT-116 cells, both *DCLK1* mRNA and protein expression were down regulated after radiation exposure (P <0. 01; Figure 1B-C).

**The inhibition of** ***DCLK1*** **enhances the sensitivity of HCT-116 cells to IR treatment**

**Fig. 2:**
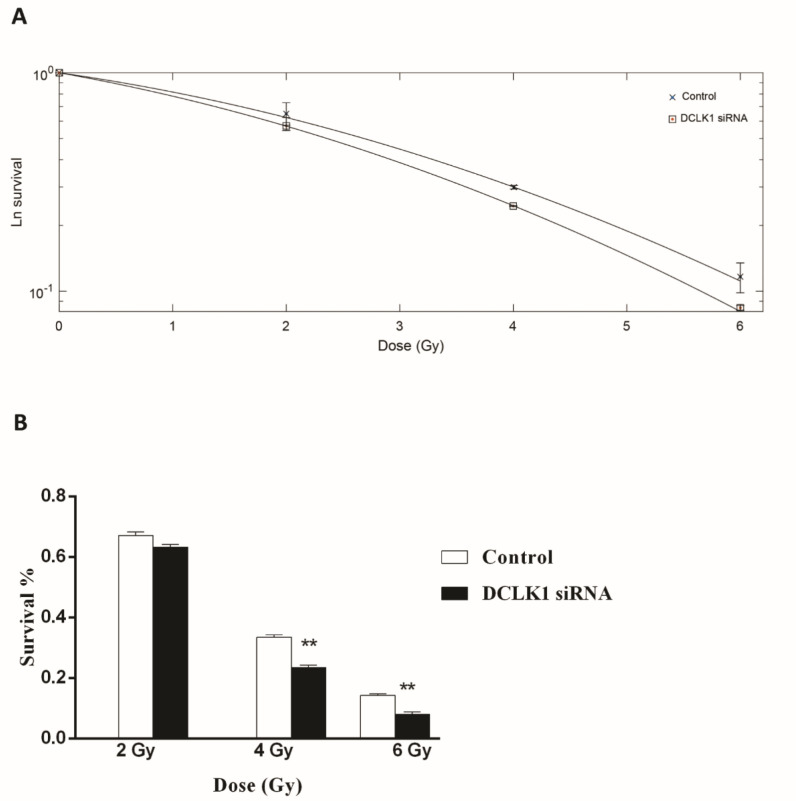
**Effect of DCLK1 silencing on the radiosensitivity of HCT-116 cells.** (A) The survival fraction of the DCLK1siRNA transfected group was lower than the untransfected group at doses of 4 and 6 Gy. (B) Representative results of colony formation of HCT-116 cells transfected with DCLK1 siRNA, and untransfected cells. **P < 0.01 *vs.* the control group

To evaluate whether the inhibition of *DCLK1 *could enhance radiosensitivity in CRC cells, a colony formation assay was performed. Compared with the control group, the survival curve of HCT-116 cells in the *DCLK1*-siRNA1 group was significantly reduced following exposure to 4, and 6 Gy doses of irradiation (P<0.01; Figure 2). Microscopic observation post crystal violet staining revealed a dose dependent decrease in the number and size of the emerged colonies in the transfected group in comparison with the non-transfected group (data not shown). Thus, our results revealed that the combined treatment of *DCLK1*-siRNA and IR led to a significant reduction in the survival fraction of HCT-116 cells compared to the IR treatment alone (Figure 2).

**Silencing** ***DCLK1*** **reduces IR-induced DNA repair**

**Fig. 3:**
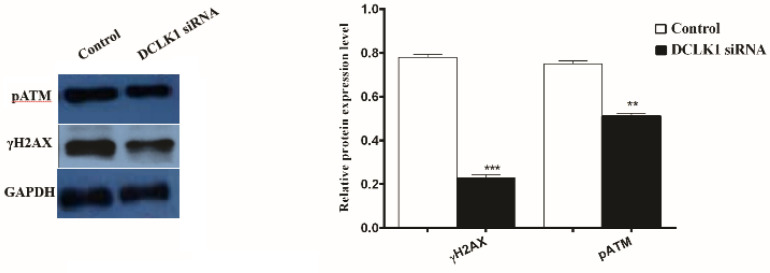
**Effect of silencing DCLK1 on DNA repair protein levels.** **‎** Western blot analysis was performed to evaluate the protein expression levels of phospho ATM (ser 1981) (pATM), γH2AX 48 and 24 h after 6 Gy X-ray in the presence and absence of DCLK, respectively. **P < 0.01 and ***P < 0.001 vs. control

To understand whether *DCLK1* expression in CRC cells regulates DNA damage response following radiation exposure, western blot analysis was performed to detect the protein expression level of pATM and phosphorylated H2AX (γH2AX) 48 and 24 h post-radiation, respectively. The results showed that ATM (P <0.01) and H2AX (*P* <0.001) phosphorylation significantly were reduced in the transfected cell in comparison with the non-transfected group (Figure 3).

***DCLK1*** **silencing prolongs IR-induced G0/G1 arrest and increases apoptosis**

**Fig. 4:**
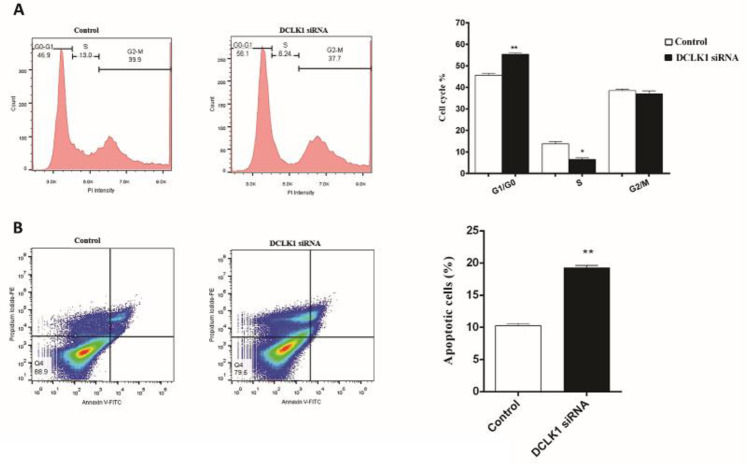
**Effect of DCLK1 inhibition on cell cycle and apoptosis.** (A) The cell cycle of transfected cells with DCLK1 siRNA was arrested in the G0/G1 phase compared with the control group ‎48 h post-exposure to 6 Gy irradiation. (B) The apoptosis was analyzed by flow cytometry. The percentage of apoptotic cells markedly increased following IR combined with DCLK1 siRNA. *P < 0.05, **P < 0.01 and ***P < 0.001 *vs.* control

Here, cell cycle analysis was conducted by flow cytometry to elucidate whether *DCLK1 *silencing combined with IR impact the cell cycle distribution of HCT-116 cells. The G0/G1 arrest of siRNA transfected cells was significantly higher at 48 h post-exposure to 6 Gy irradiation compared to the non-transfected cells (P < 0.01; Figure 4A). Therefore, it can be concluded that silencing *DCLK1* increased radiosensitivity by inducing G0/G1 arrest. 

It is well known that apoptosis is one of the major IR-induced cell death. To find out the role of *DCLK1* silencing in inducing apoptosis in HCT-116 cells after radiation exposure, apoptosis analysis was performed by flow cytometry. The apoptotic rate was significantly elevated in the *DCLK1*-siRNA treatment group 48 h post-exposure to a single dose of 6 Gy IR (P < 0.01; Figure 4B). 

**Fig. 5.**
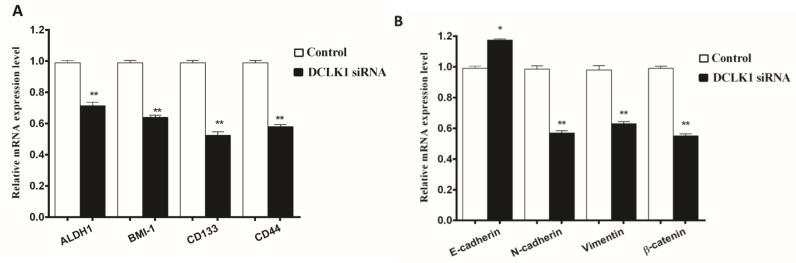
**Effects of DCLK1 siRNA combined with IR on CSCs and EMT-related markers.** (A) RT-PCR analysis of mRNA expression of CSCs markers of transfected HCT-116 cells with DCLK1 siRNA and untransfected cells 48 h after ‎6 Gy irradiation‎. Stemness factors mRNA levels were significantly lower in the DCLK1 siRNA group. (B) Cells were transfected with DCLK1 inhibitor for 24 h and treated with 6 Gy radiation. The mRNA levels of *N-cadherin*, *vimentin* and *β-catenin* were lower in cells transfected with DCLK1 siRNA. Conversely, the expression of *E-cadherin* in the absence of *DCLK1* was higher than that in the presence of *DCLK1*.*P < 0.05 and **P < 0.01 *vs.* control

***DLCK1*** **inhibition reduces CSCs and EMT related markers expression**

To determine the effect of *DCLK1* inhibition on CSCs and EMT-related markers, the expression patterns of key proteins were quantified. The expression of CSCs markers including *CD44*, prominin (*CD133*), aldehyde dehydrogenase 1 (*ALDH1*) and polycomb complex protein (*BMI1*) was lower in transfected cells in comparison with control cells (P <0.01; Figure 5A). RTqPCR was performed to determine EMT marker gene expression levels. As presented in Figure 5B, mesenchymal markers such as vimentin, N‐cadherin and β-catenin mRNA were down regulated (P < 0.01), while E-cadherin was up regulated (P < 0.05) (Figure 5A).

## Discussion

RT is a highly effective ‎cancer ‎treatment ‎in which ‎about half of all cancer patients receive this treatment modality ‎during their ‎course ‎of illness (3). Despite outstanding achievements in the treatment of CRC by radiotherapeutic procedure, nevertheless, radioresistance remains a major clinical problem (15). To overcome the radioresistance, there is an urgent need to identify the resistance effectors to suppress them and enhance the efficacy of treatment as well as to improve patient outcomes. 

DCLK1 is a microtubule-associated protein in which its expression has been reported to be critically required for maintaining the growth of human colon cancer cells (16). In the present study, *DCLK1* was up regulated in radioresistant HCT-116 cells; thereby most probably *DCLK1 *contributes to radioresistance of CRC. Very few studies have considered the relation between *DCLK1* inhibition and radiosensitivity. Here, we showed that *DCLK1* silencing is associated with IR sensitivity in CRC according to the dose–survival curve analysis, and this is consistent with the published studies that supported *DCLK1* as an oncogene (17, 18). Our results showed a reduced ATM and γH2AX activation, a decline in EMT and CSCs markers, increased apoptotic cell numbers and G0/G1 cell cycle arrest in *DCLK1* silenced cells after irradiation.

Cancer cells through the repair of therapeutically induced DNA damage are able to resist IR, thus down regulation of proteins in DNA repair pathways may boost tumor cell sensitivity to RT (19). Overexpression of DNA damage repair-related proteins is associated with increased radioresistance in several types of human cancer cells (20, 21). It is well known that in the DNA damage repair pathway, ATM as a central kinase plays a major role in controlling genome stability and cell survival (22). ATM silencing was reported to improve the therapeutic efficacy of DNA damaging agents on glioma cells and mantle cell lymphoma (5, 23). Indeed, inhibition of ATM kinase in tumor cells by preventing DNA repair, decreasing cell cycle checkpoint activation, and enhancing apoptosis led to radiosensitivity (24). In an early response to DNA damage, phosphorylation of H2AX at Ser-139 by ATM led to produce γH2AX, which creates an epigenetic signal for specific domains on downstream DNA damage repair proteins (25). Lack of H2AX is associated with genomic instability and radiation sensitivity (26). Measurement of γ-H2AX foci levels in cells provides a sensitive marker for detecting DSB repair efficiency because higher γ-H2AX expression associates with more unrepaired DSBs (27). Recently, it has been shown that *ATM* knockdown sensitized breast cancer cells to irradiation, and reduced phosphorylation of γ-H2AX, highlighting a strong relationship between ATM, DNA repair pathway and radioresistance (28). We observed that *DCLK1* down regulation resulted in a reduction of ‎pATM and γH2AX expression levels at 48 and 24 h post-radiation, respectively. These findings are consistent with the results from a study reporting the association of ATM phosphorylation with ‎*DCLK1* expression levels in intestinal tuft cells (29). Collectively, *DCLK1* expression appears to contribute to radioresistance in CRC through additional mechanisms that maintain DNA integrity after IR ‎exposure.

The sensitivity of cancer cells to IR depends on delays or arrests in G1, S, and G2 cell cycle phases (30). IR-induced DNA damage in proliferating cells promotes an arrest of mammalian cells at G0/G1 or G2/M phases (31). In the present study, there was an increase in the amount of G1 phase cells and decrease in S and G2 phases cells in transfected HCT-116 cells, 48 h after radiation. Many mediators are contributing to cell cycle arrest, such as IR-induced DNA damage which triggers G1 arrest through activation of the tumor suppressor gene *p53* (32). Also, we found that the reduction amount of S phase cells in the transfected group in comparison with the control group may be linked to the silencing of *DCLK1*, revealing an improvement in sensitivity of CRC cells to IR treatment. On the other hand, an increase in the amount of S phase cells of the control group could be related to normal activation of ATM after radiation, which causes the repair of damaged cells and cell proliferation maintenance. These findings are similar to some previous studies on various cancers such as breast and cervix (33, 34).

In the present study, we investigated the impact of *DCLK1* on EMT phenotype and CSCs markers. Our results revealed for the first time that inhibition of *DCLK1* radiosensitized CRC cells partly by modulating EMT genes. The EMT as a highly dynamic process not only regulates normal embryonic development, wound healing and tissue regeneration, but is also involved in all stages of tumorigenesis from initiation to metastatic expansion (35). Furthermore, it has been accepted that loss of epithelial and gain of mesenchymal markers in various cancers was associated with radioresistance (36). In our study, there was a reduction in N-cadherin and vimentin expression, but up regulation of E-cadherin in *DCLK1* silenced cells following radiation. Similar results were also reported in the pancreas and breast cancers (37, 38). For example, it has been indicated that sensitivity to RT was more evident in breast cancer cells expressing E-cadherin, relative to the breast cancer cells with no E-cadherin (36). However, less is known about the effect of EMT-related factors on tumor radioresistance.

It has been reported that activation of EMT increased the self-renewal and multi-differentiation potential of CSCs, and there is a close relationship between EMT and stemness factors (39). We found that the expression of CSCs markers decreased in cells transfected with *DCLK1* siRNA after radiation. CSCs have unique properties such as high DNA repair capacity, high expression of anti-apoptotic genes, and reactive oxygen species (ROS) scavengers which cause cancer radioresistance (2). For ‎example, *BMI1* conferred radioresistance to CD133-positive glioblastoma multiform via either ‎interaction with p-ATM, γH2AX, or global chromatin remodeling (40).

Accumulating evidence reveals that Wnt/β-catenin signaling as a major regulator of EMT and CSCs process may be a possible target to overcome the resistance to RT (41). β-catenin has a critical role in protecting cells from radiation-induced death through elevating DSBs repair, ROS scavenging, and suppressing apoptosis (41). In addition, the deregulation of Wnt signaling is linked with the radioresistance in numerous cancers (41, 42). It has been proven that DCLK1 protein is an essential effector in maintaining β–catenin expression for cell survival (11, 29). Similarly, we revealed that the down regulation of *DCLK1* in CRC cells reduced β–catenin expression after IR.

In summary, we found for the first time that the silencing of* DCLK1* enhanced the radiosensitivity of CRC cells. Decreased DSB repair, EMT and CSCs related genes down regulation, enhanced G0/G1 arrest and apoptosis, and suppressed β-catenin signaling, contribute to an increase in radiosensitivity induced by *DCLK1* inhibition. Therefore, the present observation suggests that the combination of* DCLK1* down regulation with ionizing radiation could serve as a promising therapeutic strategy to reverse radioresistance in CRC. Further radiobiological studies are required to highlight the role of DCLK1 and its downstream signaling pathway with radiosensitivity of other tumor cell lines.

## References

[1] Siegel RL, Miller KD, Jemal A (2018). Cancer statistics, 2018. CA Cancer J Clin.

[2] Li F, Zhou K, Gao L (2016). Radiation induces the generation of cancer stem cells: A novel mechanism for cancer radioresistance. Oncol Lett.

[3] Baskar R, Lee KA, Yeo R (2012). Cancer and radiation therapy: current advances and future directions. Int J Med Sci.

[4] Tang L, Wei F, Wu Y (2018). Role of metabolism in cancer cell radioresistance and radiosensitization methods. J Exp Clin Cancer Res.

[5] Li Y, Li L, Li B (2016). Silencing of ataxia-telangiectasia mutated by siRNA enhances the in vitro and in vivo radiosensitivity of glioma. Oncol Rep.

[6] Liu Y, Chen X, Hu Q (2018). Resistance to radiotherapy in lung cancer. Int J Clin Exp Med.

[7] Murata K, Saga R, Monzen S (2019). Understanding the mechanism underlying the acquisition of radioresistance in human prostate cancer cells. Oncol Lett.

[8] Chaiswing L, Weiss HL, Jayswal RD (2018). Profiles of Radioresistance Mechanisms in Prostate Cancer. Crit Rev Oncog.

[9] Samadi P, Afshar S, Amini R (2019). Let-7e enhances the radiosensitivity of colorectal cancer cells by directly targeting insulin-like growth factor 1 receptor. J Cell Physiol.

[10] Shu T, Tseng HC, Sapir T (2006). Doublecortin-like kinase controls neurogenesis by regulating mitotic spindles and M phase progression. Neuron.

[11] Chandrakesan P, Weygant N, May R (2014). DCLK1 facilitates intestinal tumor growth via enhancing pluripotency and epithelial mesenchymal transition. Oncotarget.

[12] Shibue T, Weinberg RA (2017). EMT, CSCs, and drug resistance: the mechanistic link and clinical implications. Nat Rev Clin Oncol.

[13] Khoshinani HM, Afshar S, Pashaki AS (2017). Involvement of miR-155/FOXO3a and miR-222/PTEN in acquired radioresistance of colorectal cancer cell line. Jpn J Radiol.

[14] Bradford MM (1976). A rapid and sensitive method for the quantitation of microgram quantities of protein utilizing the principle of protein-dye binding. Anal Biochem.

[15] Geng L, Wang J (2017). Molecular effectors of radiation resistance in colorectal cancer. Precis Radiat Oncol.

[16] Sarkar S, Popov VL, O'Connell MR (2017). A novel antibody against cancer stem cell biomarker, DCLK1-S, is potentially useful for assessing colon cancer risk after screening colonoscopy. Lab Invest.

[17] Ali N, Chandrakesan P, Nguyen CB (2015). Inflammatory and oncogenic roles of a tumor stem cell marker doublecortin-like kinase (DCLK1) in virus-induced chronic liver diseases. Oncotarget.

[18] Ali N, Nguyen CB, Chandrakesan P (2020). Doublecortin-like kinase 1 promotes hepatocyte clonogenicity and oncogenic programming via non-canonical β-catenin-dependent mechanism. Sci Rep.

[19] Gavande NS, VanderVere-Carozza PS, Hinshaw HD (2016). DNA repair targeted therapy: The past or future of cancer treatment?. Pharmacol Ther.

[20] Zhang P, Wei Y, Wang L (2014). ATM-mediated stabilization of ZEB1 promotes DNA damage response and radioresistance through CHK1. Nat Cell Biol.

[21] Wang W, Guo M, Xia X (2017). XRRA1 Targets ATM/CHK1/2-Mediated DNA Repair in Colorectal Cancer. Biomed Res Int.

[22] Cremona CA, Behrens A (2014). ATM signalling and cancer. Oncogene.

[23] Golla RM, Li M, Shen Y (2012). Inhibition of poly (ADP‐ribose) polymerase (PARP) and ataxia telangiectasia mutated (ATM) on the chemosensitivity of mantle cell lymphoma to agents that induce DNA strand breaks. Hematol Oncol.

[24] Raleigh DR, Haas-Kogan DA (2013). Molecular targets and mechanisms of radiosensitization using DNA damage response pathways. Future Oncol.

[25] Mah LJ, El-Osta A, Karagiannis TC (2010). gammaH2AX: a sensitive molecular marker of DNA damage and repair. Leukemia.

[26] Banath JP, Macphail SH, Olive PL (2004). Radiation sensitivity, H2AX phosphorylation, and kinetics of repair of DNA strand breaks in irradiated cervical cancer cell lines. Cancer Res.

[27] Lee Y, Wang Q, Shuryak I (2019). Development of a high-throughput γ-H2AX assay based on imaging flow cytometry. Radiat Oncol.

[28] Bian L, Meng Y, Zhang M (2020). ATM Expression Is Elevated in Established Radiation-Resistant Breast Cancer Cells and Improves DNA Repair Efficiency. Int J Biol Sci.

[29] Chandrakesan P, May R, Weygant N (2016). Intestinal tuft cells regulate the ATM mediated DNA Damage response via Dclk1 dependent mechanism for crypt restitution following radiation injury. Sci Rep.

[30] Bernhard EJ, Maity A, Muschel RJ (1995). Effects of ionizing radiation on cell cycle progression. A review. Radiat Environ Biophys.

[31] Pawlik TM, Keyomarsi K (2004). Role of cell cycle in mediating sensitivity to radiotherapy. Int J Radiat Oncol Biol Phys.

[32] Levine AJ, Oren M (2009). The first 30 years of p53: growing ever more complex. Nat Rev Cancer.

[33] Chuang JY, Tsai YY, Chen SC (2005). Induction of G0/G1 arrest and apoptosis by 3-hydroxycinnamic acid in human cervix epithelial carcinoma (HeLa) cells. In Vivo.

[34] Murad H, Hawat M, Ekhtiar A (2016). Induction of G1-phase cell cycle arrest and apoptosis pathway in MDA-MB-231 human breast cancer cells by sulfated polysaccharide extracted from Laurencia papillosa. Cancer Cell Int.

[35] Roche J, Erratum: Roche, J (2018). The Epithelial-to-Mesenchymal Transition in Cancer. Cancers. Cancers (Basel).

[36] Theys J, Jutten B, Habets R (2011). E-Cadherin loss associated with EMT promotes radioresistance in human tumor cells. Radiother Oncol.

[37] Liu H, Wen T, Zhou Y (2019). DCLK1 Plays a Metastatic-Promoting Role in Human Breast Cancer Cells. Biomed Res Int.

[38] Ikezono Y, Koga H, Akiba J (2017). Pancreatic Neuroendocrine Tumors and EMT Behavior Are Driven by the CSC Marker DCLK1. Mol Cancer Res.

[39] Weidenfeld K, Barkan D (2018). EMT and Stemness in Tumor Dormancy and Outgrowth: Are They Intertwined Processes?. Front Oncol.

[40] Facchino S, Abdouh M, Chatoo W (2010). BMI1 confers radioresistance to normal and cancerous neural stem cells through recruitment of the DNA damage response machinery. J Neurosci.

[41] Zhao Y, Tao L, Yi J (2018). The Role of Canonical Wnt Signaling in Regulating Radioresistance. Cell Physiol Biochem.

[42] Zhao Y, Yi J, Tao L (2018). Wnt signaling induces radioresistance through upregulating HMGB1 in esophageal squamous cell carcinoma. Cell Death Dis.

